# Molecular detection of *Histoplasma capsulatum* in organ samples from bats randomly captured in urban areas of Araraquara, São Paulo state, Brazil

**DOI:** 10.1017/S0950268824000657

**Published:** 2024-05-06

**Authors:** Jessica A. Ruiz-Muñoz, Gabriela Rodríguez-Arellanes, José A. Ramírez, Laura E. Carreto-Binaghi, Ana M. Fusco-Almeida, Maria J. S. Mendes-Giannini, Blanca E. García-Pérez, Maria L. Taylor

**Affiliations:** 1 Unidad de Micología, Departamento de Microbiología-Parasitología, Facultad de Medicina, Universidad Nacional Autónoma de México (UNAM), Mexico City, Mexico; 2 Instituto Nacional de Enfermedades Respiratorias Ismael Cosío Villegas (INER), Mexico City, Mexico; 3 Departamento de Análises Clínicas, Faculdade de Ciências Farmacêuticas, Universidade Estadual Paulista (UNESP), São Paulo, Brazil; 4 Departamento de Microbiología, Escuela Nacional de Ciencias Biológicas, Instituto Politécnico Nacional (IPN), Mexico City, Mexico

**Keywords:** bats, *H. capsulatum*, Hcp100 marker, histoplasmosis, nested PCR

## Abstract

The mycosis histoplasmosis is also considered a zoonosis that affects humans and other mammalian species worldwide. Among the wild mammals predisposed to be infected with the etiologic agent of histoplasmosis, bats are relevant because they are reservoir of *Histoplasma* species, and they play a fundamental role in maintaining and spreading fungal propagules in the environments since the infective mycelial phase of *Histoplasma* grows in their accumulated guano. In this study, we detected the fungal presence in organ samples of bats randomly captured in urban areas of Araraquara City, São Paulo, Brazil. Fungal detection was performed using a nested polymerase chain reaction to amplify a molecular marker (Hcp100) unique to *H. capsulatum*, which revealed the pathogen presence in organ samples from 15 out of 37 captured bats, indicating 40.5% of infection. Out of 22 Hcp100-amplicons generated, 41% corresponded to lung and trachea samples and 59% to spleen, liver, and kidney samples. Data from these last three organs suggest that bats develop disseminated infections. Considering that infected bats create environments with a high risk of infection, it is important to register the percentage of infected bats living in urban areas to avoid risks of infection to humans, domestic animals, and wildlife.

## Introduction

*Histoplasma capsulatum*, the etiologic agent of systemic mycosis histoplasmosis, is a dimorphic ascomycete found in nature as mycelial morphotype (infective M-phase). Throughout the infection in the host, the M-phase converts to its yeast morphotype (parasitic Y-phase). In humans, it can evolve as a life-threatening disease with manifestations ranging from mild-to-severe disseminated clinical forms. In the environment, this fungus grows in soil supplemented with bat and bird guano, which contains high concentrations of nitrogen, phosphorous, and other oligoelements [1, 2]. These micronutrients along with particular physical conditions such as darkness, temperature between 25–30 °C, and relative humidity >60%, constitute the ideal ecological niche for the development of this pathogenic microorganism [3].

Infected wild mammals, mainly bats, as well as some birds and microarthropods species, can act as fungal dispersers in the environment [4–6]. This dispersion activity is related to special ecological niches, which are shared by the fungus and their dispersers either in enclosed spaces (caverns, caves, abandoned mines, etcetera) or in open spaces (public parks, streets, houses, uninhabited buildings, etcetera). Due to their colonial behaviour, colony size, and ability to fly, bats are considered the main *H. capsulatum* disperser. They facilitate fungal growth in wild and urban areas where several bats species maintain their shelters, which constitute places with a high risk of infection. Some reports have documented *H. capsulatum* isolation from infected bats randomly captured in urban shelters or the fungal presence in the environments of urban buildings, producing a risk of outbreaks [7–11].

It has also been reported that randomly captured bats can develop a disseminated histoplasmosis infection, as evidenced by positive *H. capsulatum* cultures obtained from their liver and spleen [3], which are crucial organs of the mononuclear phagocytic system. In addition, Suárez-Álvarez et al. [12] reported the presence of intracellular yeasts in the nasal-associated lymphoid tissue (NALT), the nasal mucosa non-associated with the NALT and within the interdigitating dendritic cells of the cervical lymph node, 2 hours after bats and mice were intranasally infected with *H. capsulatum* mycelial propagules, which suggests early fungal dimorphism and dissemination via the lymph vessels. Therefore, these findings support the possible role of bats in maintaining *Histoplasma* species in nature, by acting as a reservoir.

Autochthonous outbreaks in extreme latitudes, such as 54°N in Alberta (Canada) and 38°S in the Argentinian Patagonia [13, 14], support a major spreading of the pathogen in the environment [4]. Undoubtedly, the geographical distribution of *H. capsulatum* has been amplified, possibly due to changes in the migratory route of its natural dispersers. This fact can be explained by new occurrences associated with climatic changes that generate adaptation processes either in the pathogen or in their dispersers and reservoirs [4].

The *Histoplasma* taxonomy is under rearrangement. New information about the *H. capsulatum* classification has been proposed by Sepúlveda et al. [15], who used a phylogenomic approach. These authors renewed the names of some phylogeographical clusters previously identified by Kasuga et al. [16], by renaming the lineage H81 from Panama as well as the clusters NAm 1, NAm 2, and LAm A, such as *H. capsulatum* sensu stricto Darling 1906, *H. mississippiense* sp. nov., *H. ohiense* sp. nov., and *H. suramericanum* sp. nov., respectively. However, based on the data from Kasuga et al. [16], Teixeira et al. [17], Rodrigues et al. [18], and Vite-Garín et al. [19], our research team has reconsidered that *H. capsulatum* has at least 14 phylogenetic groups and four lone lineages [4].

The infection and the clinical course of histoplasmosis begin by inhaling M-phase propagules (mainly microconidia and small hyphal fragments), following the respiratory route with a further establishment in the lungs. This process could be modified by the inoculum size, virulence, and phylogenetic species of the fungal pathogen, as well as by the host’s immune status (mainly in immunosuppressive conditions). Transition to the Y-phase is an absolute requirement for the progression of histoplasmosis, as demonstrated by pathogenic strains treated with p-chloromercuriphenylsulfonic (PCMPS) acid, which generate a strain irreversibly altered that was unable to produce experimental histoplasmosis in mice inoculated with PCMPS acid-treated M-phase [20].

Thus, for a successful infection, a fast M- to Y-phase transition is necessary, which is crucial for the infection dissemination and the pathogenesis of histoplasmosis [12, 20, 21]. Overall, fungal dissemination occurs through the lymphatic and blood vessels [12]. Several data suggest that bats develop an effective defence mechanism against *H. capsulatum* infection, which is characterized by a few inflammatory reactions in the infected tissues [3]. Thus, the lack of severity in their natural infection is mystifying up-to-date.

Considering the critical role of bats in the interaction with the pathogen *H. capsulatum*, this study aimed to evidence a dangerous presence of infected bats on the borders of urban areas, which could represent a risk factor for fungal infection in humans living near their shelters.

## Materials and methods

### Bats

We studied 37 bats from different species, which were randomly captured with a mist-net in urban areas of Araraquara City, Araraquara municipality, São Paulo state, Brazil. They were captured, transported, and immediately processed by researchers at the Laboratorio de Micología Clínica, Faculdade de Ciências Farmacêuticas, Campus Araraquara, UNESP, São Paulo, Brazil. The trachea, lungs, spleen, liver, kidney, and intestines were aseptically removed. These organ samples were preserved in 70% ethanol and sent to the Laboratorio de Inmunología de Hongos, Unidad de Micología, Facultad de Medicina, UNAM, Ciudad de México, CDMX, Mexico, where the subsequent molecular detection of *H. capsulatum* was performed. The international guidelines published in the Guide for the Care and Use of Laboratory Animals of the National Research Council (US) Committee [22], were strictly followed during the capture of bats and their processing in the laboratory. Table 1 summarizes the most relevant information of each bat specimen studied.

**Table 1.** Data from the studied bats

Bat registers in Mexico/Brazil	Bat species	Gender	Capture date/Capture site
M–559/802	*Eumops auripendulus*	Female	11–07–2016/House yard
M–560/805*	*Eumops perotis*	Male	15–07–2016/House garden
M–561/807	*Molossus molossus*	Male	22–09–2016/House yard
M–562/810	*E. auripendulus*	Male	05–09–2016/House
M–563/821	*M. molossus*	Male	19–04–2016/House
M–564/822	*M. molossus*	Male	25–04–2016/House yard
M–565/825	*E. auripendulus*	Female	29–04–2016/House yard
M–566/828	*E. auripendulus*	Female	16–05–2016/House
M–567/832	*Glossophaga soricina*	Male	08–03–2016/House
M–568/835	*E. auripendulus*	Female	16–03–2016/House yard
M–569/841	*No italic*	Female	01–04–2016/Street
M–570/845	*E. auripendulus*	Female	04–04–2016/House
M–571/854*	*M. rufus*	Female	18–01–2016/House garden
M–572/857	*E. auripendulus*	Female	01–01–2016/Street
M–573/860	*E. auripendulus*	Female	21–01–2016/Street
M–574/861	*E. auripendulus*	Female	21–01–2016/Street
M–575/862*	*M. molossus*	Female	02–02–2016/Street
M–576/865	*E. auripendulus*	Female	10–02–2016/House
M–577/868	*M. molossus*	Male	19–04–2016/House
M–578/870	*M. rufus*	Female	22–02–2016/House
M–579/888	*Artibeus lituratus*	Female	18–01–2016/House
M–580/905	*Tadarida brasiliensis*	Male	05–12–2016/House yard
M–581/908	*E. auripendulus*	Male	05–12–2016/House yard
M–582/912*	*E. perotis*	Female	12–12–2016/Street
M–583/914*	*E. auripendulus*	Male	12–12–2016/House yard
M–584/932	*E. perotis*	Female	06–02–2017/House yard
M–585/934	*M. molossus*	Male	07–02–2017/House yard
M–586/955	*E. auripendulus*	Male	12–12–2016/House
M–587/958	*M. molossus*	Male	12–12–2016/House
M–588/960	*M. molossus*	Male	12–12–2016/House
M–589/961	*E. auripendulus*	Female	17–10–2016/Street
M–590/962*	*M. rufus*	Male	20–10–2016/House yard
M–591/964*	*E. auripendulus*	Male	09–11–2016/House yard
M–592/972	*A. lituratus*	Female	08–09–2016/Street
M–593/974	*E. auripendulus*	Female	22–09–2016/House yard
M–594/976	*E. auripendulus*	Male	22–09–2016/House yard
M–595/978	*M. rufus*	Male	23–09–2016/Street

* Captured dead.

### DNA extraction from bat organs

Each bat organ sample was used for DNA extraction using a commercial DNeasy Blood & Tissue Kit (Qiagen, Valencia, CA, USA), following the manufacturer’s instructions. DNA samples were quantified in an Epoch microplate spectrophotometer (BioTek Instruments Inc., Winooski, VT, USA) at 260–280 nm. All DNA samples were processed in a single cabinet for each step of the molecular assay, and DNA manipulation was conducted with sterile maximum recovery pipet tips and microtubes (Axygen Scientific Inc., Union City, CA, USA). DNA samples were frozen and stored at −20 °C, until required.

### Detection of *H. capsulatum* by nested polymerase chain reaction of the DNA extracted from each bat organ

DNA samples were screened for *H. capsulatum* infection using a nested polymerase chain reaction (PCR) to amplify a highly specific fragment of the gene encoding a 100-kDa protein (Hcp100), as reported by Bialek et al. [23]. This molecular marker is considered unique to this pathogen. The first and second (nested) PCR reactions were conducted according to Bialek et al. [23], including minor modifications suggested by González-González et al. [24], which did not change the specificity and sensitivity of the Hcp100 marker. Two sets of primers were used: the outer primer set included the HcI (5′-GCG TTC CGA GCC TTC CAC CTC AAC-3′) and HcII (5′-ATG TCC CAT CGG GCG CCG TGT AGT-3′) primers, which delimit a 391-base pair (bp) gene fragment in the first PCR reaction; the inner primer set included the HcIII (5′-GAG ATC TAG TCG CGG CCA GGT TCA-3′) and HcIV (5′-AGG AGA GAA CTG TAT CGG TGG CTT G-3′) primers, which delimit a 210 bp fragment specific to *H. capsulatum* in the nested reaction.

The primers were supplied by Operon Technologies Inc. (Alameda, CA, USA). The first and nested reactions of the *Hcp100* gene fragment were standardized according to González-González et al. [24], including minor modifications. For the first round of amplification, the thermocycling conditions were as follows: one cycle at 94 °C for 5 min; 30 cycles at 94 °C for 30 s, 50 °C for 30 s, and 72 °C for 1 min; and a final cycle at 72 °C for 5 min. For the nested reaction, the thermocycling conditions were: one cycle at 95 °C for 5 min; 30 cycles at 94 °C for 30 s, 65 °C for 30 s, and 72 °C for 1 min; and a final extension cycle at 72 °C for 10 min. The DNA from the EH-46 *H. capsulatum* strain, a reference strain from our laboratory, was used as a control for positive amplification and milli-Q water was always processed as a negative control.

### Amplified products and sequencing

Amplicons of the nested reaction were electrophoresed on 1.5% agarose in 0.5X Tris-borate-EDTA buffer at 100 V for 80 min; using as a molecular size marker 1.5 μl of the 100 bp DNA Ladder (New England Biolabs, Ipswich, MA, USA) diluted 1:10. The bands were visualized using a UV transilluminator after gel red staining (0.5 μg/ml). According to González-González et al. [24], the production of amplicons for the *Hcp100* gene fragment was the main inclusion criterion considered for *H. capsulatum* bat infection. The amplicons were purified and sequenced at Macrogen Corp. (Rockville, MD, USA). Sequencing reactions were performed for forward and reverse DNA strands.

### Analysis of *H. capsulatum* sequences using the basic local alignment search tool nucleotide algorithm

The sequences were edited and aligned with the MEGA software version 5 (http://www.megasoftware.net). A consensus sequence was generated for each amplified product from the different bat organ samples, using Chromas version 2.6.5 (technelysium.com.au/wp/) and BioEdit version 5.0.9 [25].

The generated sequences aligned from 2,345 to 2,500 nucleotide (containing 156 nt) were chosen to perform the basic local alignment search tool nucleotide (BLASTn) analysis (blast.ncbi.nlm.nih.gov/Blast.cgi), using as reference the complete sequence of the *Hcp100* gene reported in the GenBank (accession number: AJ005963.1) for the G-217B strain, which is a reference strain of the *H. capsulatum* NAm 2 phylogenetic species.

### Statistics

The percentages of *H. capsulatum* infection in the bats studied were estimated for bat species, gender, their capture sites, and sampled organs. The corresponding 95% confidence intervals (CI) were calculated according to Clopper-Pearson binomial distribution, using Epitools-Epidemiological Calculations [26].

## Results

Data enlisted in Table 1 indicate the registers of the studied bats, specifying their species, gender, capture date, and capture site.

### Molecular detection of *H. capsulatum* in bat organ samples

From the 37 captured bats, only 82 DNA samples were obtained in the required amounts for processing by nested PCR. The samples generated 22 amplicons of the Hcp100 marker: two from the trachea; seven from the lungs; two from the spleen; eight from the liver; and three from the kidney (see details in Table 2).

**Table 2.** Hcp100 Nested-PCR results from the studied bats

	Sampled organs processed for Hcp100 detection					
Bats	Trachea	Lungs	Spleen	Liver	Kidney	Intestines
M–559/802	(−)	NP	(−)	NP	NP	NP
M–560/805	(−)	NP	NP	(−)	NP	NP
M–561/807	(−)	(−)	NP	(−)	NP	NP
M–562/810	(−)	(−)	NP	(−)	NP	NP
M–563/821	NP	NP	(−)	(−)	NP	NP
M–564/822	NP	(−)	(−)	(−)	NP	NP
M–565/825	NP	(−)	NP	(−)	NP	NP
M–566/828	(−)	(−)	NP	NP	NP	NP
M–567/832	NP	NP	(−)	(−)	NP	NP
M–568/835	(−)	A	NP	A	NP	NP
M–569/841	NP	(−)	(−)	(−)	NP	NP
M–570/845	NP	NP	(−)	A	NP	NP
M–571/854	NP	(−)	NP	NP	NP	NP
M–572/857	NP	(−)	NP	(−)	NP	NP
M–573/860	NP	A	A	NP	NP	NP
M–574/861	NP	NP	A	(−)	NP	NP
M–575/862	NP	A	NP	NP	NP	NP
M–576/865	NP	(−)	NP	(−)	NP	NP
M–577/868	(−)	(−)	NP	NP	NP	NP
M–578/870	(−)	NP	NP	A	NP	NP
M–579/888	NP	(−)	(−)	NP	NP	NP
M–580/905	NP	(−)	NP	(−)	NP	NP
M–581/908	NP	A	NP	A	NP	NP
M–582/912	A	(−)	NP	A	NP	NP
M–583/914	NP	NP	(−)	(−)	NP	NP
M–584/932	NP	A	NP	(−)	A	NP
M–585/934	NP	(−)	NP	(−)	NP	(−)
M–586/955	A	A	NP	NP	NP	NP
M–587/958	NP	(−)	NP	(−)	NP	NP
M–588/960	NP	(−)	NP	A	NP	NP
M–589/961	(−)	NP	NP	NP	A	NP
M–590/962	NP	A	NP	A	NP	(−)
M–591/964	(−)	(−)	NP	NP	A	NP
M–592/972	NP	(−)	NP	NP	NP	NP
M–593/974	NP	(−)	NP	NP	(−)	NP
M–594/976	NP	(−)	NP	A	(−)	NP
M–595/978	NP	(−)	NP	(−)	NP	NP

Number of bats studied = 37; number of DNA samples extracted in suitable amount for processing by nested-PCR = 82; (A) number of organ samples with Hcp100 amplification = 22. (−) = Hcp100 negative; (NP) = Non- processed DNA.

Twenty-two generated amplicons were obtained from 15 infected bats out of the 37 specimens captured (see Table 2). Thus, irrespective of bat species, gender, and capture site, considering these data the percentage of *H. capsulatum* infection in the studied bats corresponded to 40.5% (95% CI 24.8–57.9).

**Figure 1.**
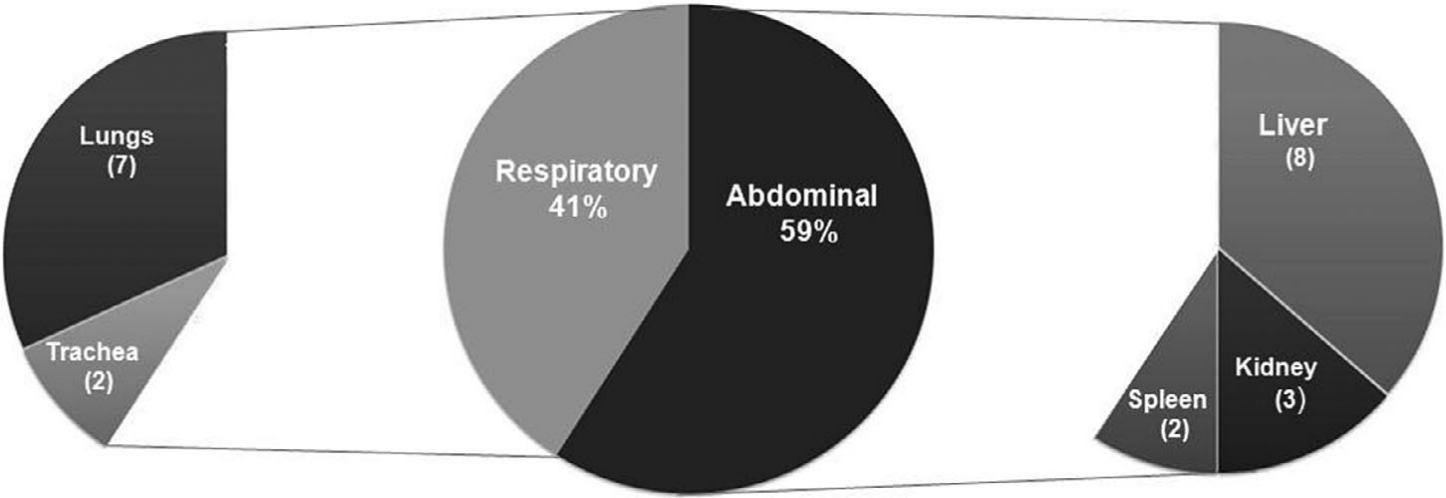
Presence of the Hcp100 marker in different organs of naturally infected bats. This figure shows the results of *H. capsulatum* infection, based on 22 amplicons generated by nested PCR for the specific marker of this fungus, highlighting the percentage of infection either in respiratory or abdominal organs. In parentheses are shown the number of positive Hcp100 amplifications either in respiratory (trachea and lungs) or abdominal (spleen, liver, and kidney) organs sampled.

Figure 1 discriminates the percentages of Hcp100 amplification by bat organs. The evidence that *H. capsulatum* infection was associated with disseminated processes was based on the 22 positive Hcp100 amplification reported in Table 2, of which 59% (95% CI 36.35–79.29) corresponded to 13 infected abdominal organs (see Figure 1), where the Hcp100 amplifications matched to three positive reactions from five kidneys, eight from 25 livers, and two from ten spleen samples processed (see Table 2). Regarding the respiratory tract, 41% (95% CI 20.71–63.65) of nine samples detected infection associated with two tracheas and seven lungs.

**Figure 2.**
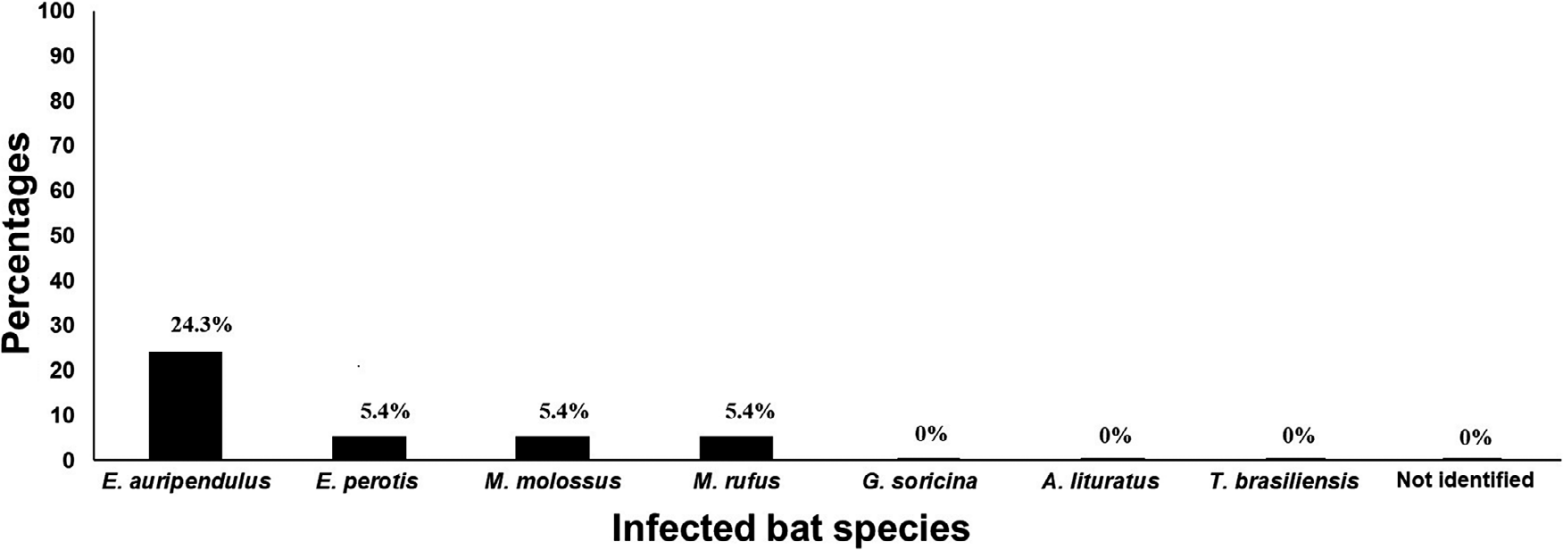
Presence of the Hcp100 marker in different infected bat species. Percentages of infected bats were calculated based on 37 studied bats. *H. capsulatum* infection data for each bat species studied was detected by the amplification of the *H. capsulatum* Hcp100 marker. See Methods section.

Based on the obtained Hcp100 sequences from organs of different bat species studied, Figure 2 shows the percentages of their *H. capsulatum* infection. Considering the 37 bats from seven well-identified bat species processed and one bat specimen non-identified taxonomically (see Table 1), data revealed that *Eumops auripendulus* presented an infection rate of 24.3% (95% CI 11.8–41.2%), followed by *E. perotis*, *Molossus molossus,* and *M. rufus* all with an infection of 5.4% (95% CI 0.7–18.2%). The remaining bat species did not show molecular evidence of fungal infection (Figure 2).

**Figure 3.**
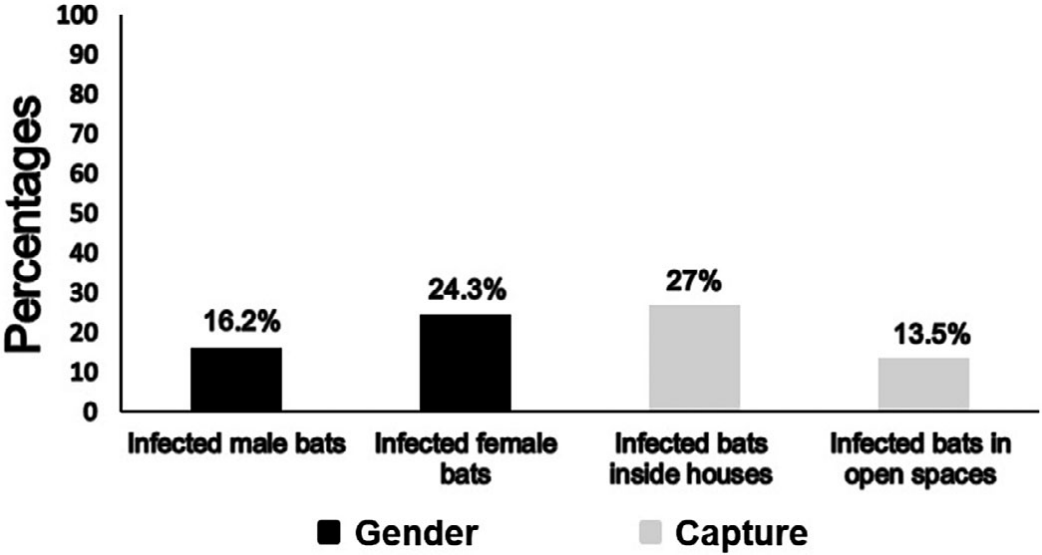
Presence of the Hcp100 marker in infected bat from different gender and capture sites. Percentages of infected bats were calculated based on 37 studied bats. *H. capsulatum* presence was identified by the amplification of the Hcp100 marker. See Methods section.

Female and male bats were captured in similar numbers (19 females and 18 males, see Table 1). Molecular data from PCR amplification from all bats studied demonstrated infections of 24.3% (95% CI 11.8–41.2%) in female and 16.2% (95% CI 6.2–32%) in male bats (see Table 2 and Figure 3).

Out of 37 studied bats, 28 were captured inside houses (yards and gardens) and nine in open spaces (see Table 1). Data from Tables 1 and 2 shows that ten bats of those captured inside houses amplified the Hcp100 fragment (27%, 95% CI 13.8–44.1%), whereas five bats of those captured in urban streets amplified this specific molecular marker (13.5%, 95% CI 4.5–28.8%) (Figure 3).

### BLASTn analysis of the *H. capsulatum* Hcp100 sequences

Although amplicons were derived from 15 infected bats (see Table 2), only 14 bats generated 19 Hcp100 sequences with accurate alignments of 156 nt that were chosen for BLASTn analysis (Table 3). For 17 out of the 19 generated sequences, the BLASTn analysis revealed high percentages of identity >90 and up to 100% (Table 3), highlighting the sequences of liver and trachea samples (M-581Lv and M-582 T), which reached 100% of identity and cover (see Table 3). However, the sequences of lung and liver samples (M-573 L and M-578Lv) only showed 85% of identity, with covers of 92 and 96%, respectively (Table 3).

**Table 3.** BLASTn analysis of the generated Hcp100 sequences

Batsamples	Cover(%)	Identity(%)	Batsamples	Cover(%)	Identity(%)
M–568 L	100	96	M–582Lv	92	98
M–568 Lv	85	94	M–584 L	94	97
M–570Lv	83	97	M–586 L	89	91
M–573 L	92	85	M–586 T	96	93
M–573S	98	100	M–588Lv	90	97
M–574S	100	98	M–589 K	98	96
M–575 L	100	98	M–590 L	98	99
M–578Lv	96	85	M–590 Lv	93	92
M–581Lv	100	100	M–594Lv	100	90
M–582 T	100	100			

K, Kidney; L, Lungs, Lv, Liver; S, Spleen; T, Trachea.

## Discussion

Generally, a high risk of histoplasmosis infection is related to inappropriate invasion of the *H. capsulatum* habitat, particularly in environments that serve as shelter for different animal species. Many mammals are susceptible to *Histoplasma* infection [3], especially bats that are considered the main reservoirs and dispersers of this fungus in the nature [3, 5, 9, 27, 28]. In this study, we revealed this fungal presence in the organs of bats randomly captured in urban areas of Araraquara City, São Paulo, Brazil, which was detected molecularly through a nested PCR for the Hcp100-specific marker of *H. capsulatum.* Thus, the existence of infected bats living in urban areas could contribute to spreading this fungus in different environments, which could be associated with a high risk for human infection. Our findings support a dangerous incidence of natural infection in bats, as suggested by Taylor et al. [3]. In addition, most bats probably developed a disseminated infection, as evidenced by the fungal presence detected molecularly, mainly in their spleen and liver, which is consistent with their role as reservoirs of *Histoplasma.*

Among the bat species captured and studied to demonstrate the presence of *Histoplasma*, both the insectivorous *E. auripendulus* and *E. perotis* species are distributed from Mexico to Brazil and Argentina, living in tropical forests, caves, tunnels, and bridges. Two other insectivorous species, *M. molossus* and *M. rufus*, are also distributed across broad zones from Mexico to Brazil and the southern areas of Uruguay and Argentina; their habitats are similar to those of the genus *Eumops* and they mainly roost in towns, abandoned buildings, and constructions sites in the cities. *Glossophaga soricina* is a species that has been recorded from Mexico to South America; they feed on insects and fruits, and their shelters are located in caves, mines, highways, buildings, and bridges. *Artibeus lituratus* is a frugivorous bat species distributed from the Isthmus of Tehuantepec, in Oaxaca, Mexico, to the north of Brazil, Argentina, Bolivia, and Paraguay, and it perches in caves, tunnels, abandoned buildings, and bridges. Finally, the insectivorous species *Tadarida brasiliensis* migrates from the centre of the United States of America at 40°N latitude to the south to Chile and Argentina at 40°S latitude; it roosts in caves, trees, silos, and abandoned buildings. All the abovementioned bat species can be found in urban and rural areas, and their summarized descriptions are based on published data in Ceballos and Oliva [29]. The distribution pattern of each bat species studied confirms their presence, either in Mexico or Brazil, in rural tropical areas where agricultural activities predominate, which have fulfilled the requirements for their feeding and reproduction. All bats species studied are colonial and share habitats among them; besides, they have also been recorded in urban areas of Mexican cities (ML Taylor, personal communication), where their accumulated guano could represent a high risk of histoplasmosis infection for immunosuppressed people living around these areas. Rural workers, whose activity is associated with bat and bird guano, as well as susceptible individuals living in urban areas, are populations at high risk of developing a histoplasmosis infection by inhaling fungal infective propagules.

For this study, it was possible to rescue 82 DNA from all bat organs sent to the Laboratorio de Inmunología de Hongos, considering that some bat samples were badly preserved during their transportation from Brazil to Mexico. Several nested PCR amplifications corresponded to lungs and trachea samples, which support the vital role of the respiratory via in fungal infection of susceptible hosts. Additionally, our results validate the information on a critical dissemination process in *H. capsulatum* infected bats, as several infected samples were retrieved from abdominal organs of the mononuclear phagocytic system, such as the spleen and liver, considering that the only natural route to enter these organs is the blood or the lymphatic vessels.

Even though the bat genus *Eumops* had an important percentage of infection, the present results did not allow proposing an association between the bat infection risks based on their species. Besides, according to our results, the most infected bat species *E. auripendulus* always showed dissemination to the spleen, liver, or kidney. Interrelated information published by Shacklette and Hasenclever [30], stated that the *H. capsulatum* infection rate changes significantly in different bats species.

Here, we expected that the occupancy of female bats in contaminated shelters with fungal infective propagules for long periods, when breeding their offspring, could lead to their high risk of infection. However, the number of infected bats in this study was not sufficient to distinguish the effect of risk of infection in regard to bats gender.

Most of the Hcp100 sequences analyzed by the BLASTn algorithm displayed high homology with the corresponding sequence deposited in the GenBank of the G-217B reference strain of *H. capsulatum.* This fact supports our criterion for confirming the presence of this pathogen in the bat organ samples.

Overall, studies about bats’ infection with *H. capsulatum* are of great relevance for generating histoplasmosis epidemiological records in urban areas and avoiding the risk of infection among the populations exposed to this pathogen distributed in different urban environments. Previous reports have registered variable rates of *H. capsulatum* infections in captured bats at urban areas in Brazil, highlighting a 34.8% of bat infection published by dos Santos et al. [31], a 20.6% described either in *Desmodus rotundus* or in *T. brasiliensis* by Veloso et al. [11], an 8.1% reported by Souza da Paz et al. [8], and a 3.6% published by Galvão-Dias et al. [7].

In conclusion, the percentages of *Histoplasma* infection in the randomly captured bats found in this study were remarkable since the accumulated bat guano could create the optimal conditions for pathogen growth in urban environments where people work, entertain, or live. Besides that, the risk of histoplasmosis infection and the development of a life-threatening disease increase in people with different immune disorders, such as Acquired Immunodeficiency Syndrome, where histoplasmosis is considered an AIDS-Defining Condition (https://www.cdc.gov/mmwr/preview/mmwrhtml/rr5710a2.html). Histoplasmosis has also been associated with the COVID-19 pandemic [32–34], although the consequences and frequency of this association are under a forthcoming evaluation process. All these facts provide enough reasons to establish preventive environmental actions in order to avoid further histoplasmosis outbreaks in high-density population areas.

## Data Availability

The data used in this study are available from the corresponding author upon reasonable request.

## References

[1] Gómez LF, et al. (2018) Detection of *Histoplasma capsulatum* in organic fertilizers by Hc100 nested polymerase chain reaction and its correlation with the physicochemical and microbiological characteristics of the samples. American Journal of Tropical Medicine and Hygiene 98, 1303–1312. 10.4269/ajtmh.17-0214. 29532772 PMC5953352

[2] Gómez-Londoño LF, et al. (2019) Capacity of *Histoplasma capsulatum* to survive the composting process. Applied and Environmental Soil Science 2019, 5038153. 10.1155/2019/5038153.

[3] Taylor ML, et al. (1999) Environmental conditions favoring bat infection with *Histoplasma capsulatum* in Mexican shelters. American Journal of Tropical Medicine and Hygiene 61, 914–919. 10.4269/ajtmh.1999.61.914. 10674670

[4] Taylor ML, et al. (2022) Considerations about the geographic distribution of the *Histoplasma* species. Applied and Environmental Microbiology 88, e0201021. 10.1128/aem.02010-21. 35262368 PMC9004366

[5] Hoff GL and Bigler WJ (1981) The role of bats in the propagation and spread of histoplasmosis: A review. Journal of Wildlife Diseases 17, 191–196. 10.7589/0090-3558-17.2.191. 7017172

[6] Estrada-Bárcenas DA, et al. (2010) Biological activity of the mite *Sancassania* sp. (Acari: Acaridae) from bat guano associated with the pathogenic fungus *Histoplasma capsulatum*. Memórias do Instituto Oswaldo Cruz - Fiocruz 105, 127–131. 10.1590/s0074-02762010000200003. 20428669

[7] Galvão-Dias MA, et al. (2011) Isolation of *Histoplasma capsulatum* from bats in the urban area of São Paulo state, Brazil. Epidemiology and Infection 139, 1642–1644. 10.1017/S095026881000289X. 21205438

[8] Souza da Paz SG, et al. (2018) Infection by *Histoplasma capsulatum*, *Cryptococcus* spp. and *Paracoccidioides brasiliensis* in bats collected in urban areas. Transboundary and Emerging Diseases 65, 1797–1805. 10.1111/tbed.12955. 30296014

[9] Taylor ML, et al. (2005) Identification of the infection source of an unusual outbreak of histoplasmosis, in a hotel in Acapulco, state of Guerrero, Mexico. FEMS Immunology and Medical Microbiology 45, 435–441. 10.1016/j.femsim.2005.05.017. 16061362

[10] da Silva JA, et al. (2021) Molecular detection of *Histoplasma capsulatum* in bats of the Amazon biome in Pará state, Brazil. Transboundary and Emerging Diseases 68, 758–766. 10.1111/tbed.13740. 32686315

[11] Salomão Veloso SC, et al. (2014) *Pneumocystis* spp. e *Histoplasma* *capsulatum* detectados em pulmões de morcegos das regiões Sul e Centro-Oeste do Brasil. Acta Scientiae Veterinariae 42, 1252.

[12] Suárez-Álvarez RO, et al. (2019) Dimorphism and dissemination of *Histoplasma capsulatum* in the upper respiratory tract after intranasal infection of bats and mice with mycelial propagules. American Journal of Tropical Medicine and Hygiene 101, 716–723. 10.4269/ajtmh.18-0788. 31287042 PMC6726946

[13] Anderson H, et al. (2006) Histoplasmosis cluster, golf course, Canada. Emerging Infectious Diseases 12, 163–165. 10.3201/eid1201.051083. 16494738 PMC3291405

[14] Calanni LM, et al. (2013) Brote de histoplasmosis en la provincia de Neuquén, Patagonia Argentina. Revista Iberoamericana de Micología 30, 193–199. 10.1016/j.riam.2012.12.007. 23402833

[15] Sepúlveda VE, et al. (2017) Genome sequences reveal cryptic speciation in the human pathogen *Histoplasma capsulatum*. MBio 8, e01339–e01317. 10.1128/mBio.01339-17. 29208741 PMC5717386

[16] Kasuga T, et al. (2003) Phylogeography of the fungal pathogen *Histoplasma capsulatum*. Molecular Ecology 12, 3383–3401. 10.1046/j.1365-294x.2003.01995.x. 14629354

[17] Teixeira MM, et al. (2016) Worldwide phylogenetic distributions and population dynamics of the genus *Histoplasma*. PLoS Neglected Tropical Diseases 10, e0004732. 10.1371/journal.pntd.000473227. 27248851 PMC4889077

[18] Rodrigues AM, et al. (2020) The global epidemiology of emerging *Histoplasma* species in recent years. Studies in Mycology 97, 100095. 10.1016/j.simyco.2020.02.001. 33335607 PMC7714791

[19] Vite-Garín T, et al. (2021) *Histoplasma capsulatum* isolated from *Tadarida brasiliensis* bats captured in Mexico form a sister group to north American class 2 clade. Journal of Fungi 7, 529. 10.3390/jof7070529. 34209122 PMC8305335

[20] Medoff G, et al. (1986) Irreversible block of the mycelial-to-yeast phase transition of *Histoplasma capsulatum*. Science 231, 476–479. 10.1126/science.3001938. 3001938

[21] Sahaza JH, et al. (2014) Usefulness of the murine model to study the immune response against *Histoplasma capsulatum* infection. Comparative Immunology, Microbiology and Infectious Diseases 37, 143–152. 10.1016/j.cimid.2014.03.002. 24766724

[22] National Research Council (2011) Science, Medicine, and Animals. Washington, DC: The National Academies Press, p. 246. 10.17226/10733.

[23] Bialek R, et al. (2001) Diagnosis and monitoring of murine histoplasmosis by a nested PCR assay. Journal of Clinical Microbiology 39, 1506–1509. 10.1128/JCM.39.4.1506-1509.2001. 11283078 PMC87961

[24] González-González AE, et al. (2012) An *Hcp100* gene fragment reveals *Histoplasma capsulatum* presence in lungs of *Tadarida brasiliensis* migratory bats. Epidemiology and Infection 140, 1955–1963. 10.1017/S0950268811002585. 22152724

[25] James WB (2005) BioEdit: Biological sequence alignment editor. Version 5.0.9. Available at http://www.mbio.ncsu.edu/bioedit/bioedit.html (accessed 8 November 2023).

[26] Epitools-Epidemiological Calculations. Available at https://epitools.ausvet.com.au/ciproportion (accessed 21 November 2023).

[27] Taylor ML, Chávez-Tapia CB and Reyes-Montes MR (2000) Molecular typing of *Histoplasma capsulatum* isolated from infected bats, captured in Mexico. Fungal Genetics and Biology 30, 207–212. 10.1006/fgbi.2000.1219. 11035942

[28] Taylor ML, et al. (2005) Geographical distribution of genetic polymorphism of the pathogen *Histoplasma capsulatum* isolated from infected bats, captured in a central zone of Mexico. FEMS Immunology and Medical Microbiology 45, 451–458. 10.1006/fgbi.2000.1219. 16061361

[29] Ceballos G and Oliva G (eds.) (2005) *Los Mamíferos Silvestres de México.* Ciudad de México, MX; Comisión Nacional para el Conocimiento y Uso de la Biodiversidad (CONABIO) and Fondo de Cultura Económica (FCE), 986 pp.

[30] Shacklette MH and Hasenclever HF (1969) Variation of rates of natural infection with *Histoplasma capsulatum* in bats. American Journal of Tropical Medicine and Hygiene 18, 53–57. 10.4269/ajtmh.1969.18.53. 5764198

[31] dos Santos B, et al. (2018) Molecular detection of *Histoplasma capsulatum* in insectivorous and frugivorous bats in southeastern Brazil. Medical Mycology 56, 937–940. 10.1093/mmy/myx138. 29294049

[32] Basso RP, et al. (2021) COVID-19-associated histoplasmosis in an AIDS patient. Mycopathologia 186, 109–112. 10.1007/s11046-020-00505-1. 33156463 PMC7644795

[33] Frías-de-León MG, et al. (2021) Epidemiology of systemic mycoses in the COVID-19 pandemic. Journal of Fungi 7, 556. 10.3390/jof7070556. 34356935 PMC8307417

[34] Roudbary M, et al. (2021) Overview on the prevalence of fungal infections, immune response, and microbiome role in COVID-19 patients. Journal of Fungi 7, 720. 10.3390/jof7090720. 34575758 PMC8466761

